# Involvement of the zebrafish *trrap* gene in craniofacial development

**DOI:** 10.1038/s41598-021-03123-z

**Published:** 2021-12-21

**Authors:** Taichi Suzuki, Yo Hirai, Tomoko Uehara, Rie Ohga, Kenjiro Kosaki, Atsuo Kawahara

**Affiliations:** 1 Laboratory for Developmental Biology, Graduate School of Medical Science, University of Yamanashi, 1110 Shimokato, Chuo, Yamanashi 409-3898 Japan; 2 Center for Medical Genetics, Keio University School of Medicine, 35 Shinanomachi, Shinjuku-ku, Tokyo, 160-8582 Japan; 3 Present Address: Department of Clinical Genetics, Central Hospital, Adachi Developmental Disability Center, Aichi, Japan

**Keywords:** Developmental biology, Genetics

## Abstract

Trrap (transformation/transcription domain-associated protein) is a component shared by several histone acetyltransferase (HAT) complexes and participates in transcriptional regulation and DNA repair; however, the developmental functions of Trrap in vertebrates are not fully understood. Recently, it has been reported that human patients with genetic mutations in the *TRRAP* gene show various symptoms, including facial dysmorphisms, microcephaly and global developmental delay. To investigate the physiological functions of Trrap, we established *trrap* gene-knockout zebrafish and examined loss-of-function phenotypes in the mutants. The *trrap* zebrafish mutants exhibited smaller eyes and heads than the wild-type zebrafish. The size of the ventral pharyngeal arches was reduced and the mineralization of teeth was impaired in the *trrap* mutants. Whole-mount in situ hybridization analysis revealed that *dlx3* expression was narrowly restricted in the developing ventral pharyngeal arches, while *dlx2b* expression was diminished in the *trrap* mutants. These results suggest that *trrap* zebrafish mutants are useful model organisms for a human disorder associated with genetic mutations in the human *TRRAP* gene.

## Introduction

Trrap is a common component of various histone acetyltransferase (HAT) complexes and participates in transcription and DNA repair by recruiting HAT complexes to chromatin^1^; however, the physiological functions of TRRAP are not fully understood. Trrap possesses FAT (FRAP, ATM, TRRAP), PIKK-TRRAP (pseudokinase domain of TRRAP) and FATC (FRAP, ATM, TRRAP C-terminal) domains. The kinase domain of PIKK-TRRAP lacks catalytic activity and the functions of the FAT and FATC domains are not fully understood. *Trrap*-knockout mice exhibit peri-implantation lethality due to blocked proliferation of blastocytes^2^, and conditional disruption of the *Trrap* gene achieved by crossing *Trrap*-floxed mice and *Nestin*-Cre mice causes premature differentiation of neural progenitors in the mouse brain^3^. Disruption of the mouse *Trrap* gene in embryonic stem cells (ESCs) causes unscheduled differentiation^4^, suggesting contributions of Trrap to self-renewal and appropriate differentiation. These results suggest that Trrap plays important roles in the generation of various organs during early vertebrate embryogenesis.

In a recent study, patients with missense variants in the human *TRRAP* gene predominantly presented with facial dysmorphisms (19/24 individuals), global developmental delay (24/24 individuals) and intellectual disability (17/20 individuals)^5^. Other phenotypes, such as microcephaly (7/24 individuals), hearing impairment (3/24 individuals) and visual impairment (4/24 individuals), were also observed in the patients, who presented various clinical spectra associated with TRRAP pathogenic missense variants. RNA sequencing analysis revealed that skin fibroblasts from patients exhibit significant expression differences in several genes, suggesting the involvement of TRRAP in the transcriptional regulation of various genes. Another group independently showed that the human *TRRAP* gene is responsible for autosomal dominant nonsyndromic hearing loss (ADNSHL)^6^. The p.Arg171Cys variant of the human *TRRAP* gene cosegregated with hearing loss in ADNSHL. The authors demonstrated that knockdown using an antisense morpholino targeting the *trrap* start codon or knockout via a 7-bp deletion near the *trrap* start codon (frameshift mutation) caused a decreased number of posterior lateral line (PLL) neuromasts and an impaired acoustic startle response (ASR) in zebrafish, but they did not describe other morphological abnormalities in their *trrap* mutants. Because patients with human *TRRAP* missense mutations exhibit multiple malformations, the physiological and developmental functions of Trrap remain unclear.

Zebrafish are useful animal models with which to investigate the physiological and pathological functions of genes responsible for human disorders^7^. Zebrafish mutants for a human disease can be established to analyze the processes of morphological abnormalities during early embryogenesis. Importantly, organogenesis, including craniofacial morphogenesis, is well conserved between zebrafish and humans^8^. In this study, we established a zebrafish *trrap* mutant and investigated loss-of-function phenotypes in the heads of the mutant fish. Our findings demonstrated that the *trrap*-mutant zebrafish exhibited impairment of tooth mineralization and abnormally small eyes and head, and short ventral pharyngeal arches, which may be associated with the craniofacial abnormalities in human patients with *TRRAP* genetic mutations.

## Results

### Developmental expression of the *trrap* gene in zebrafish

**Figure 1:**
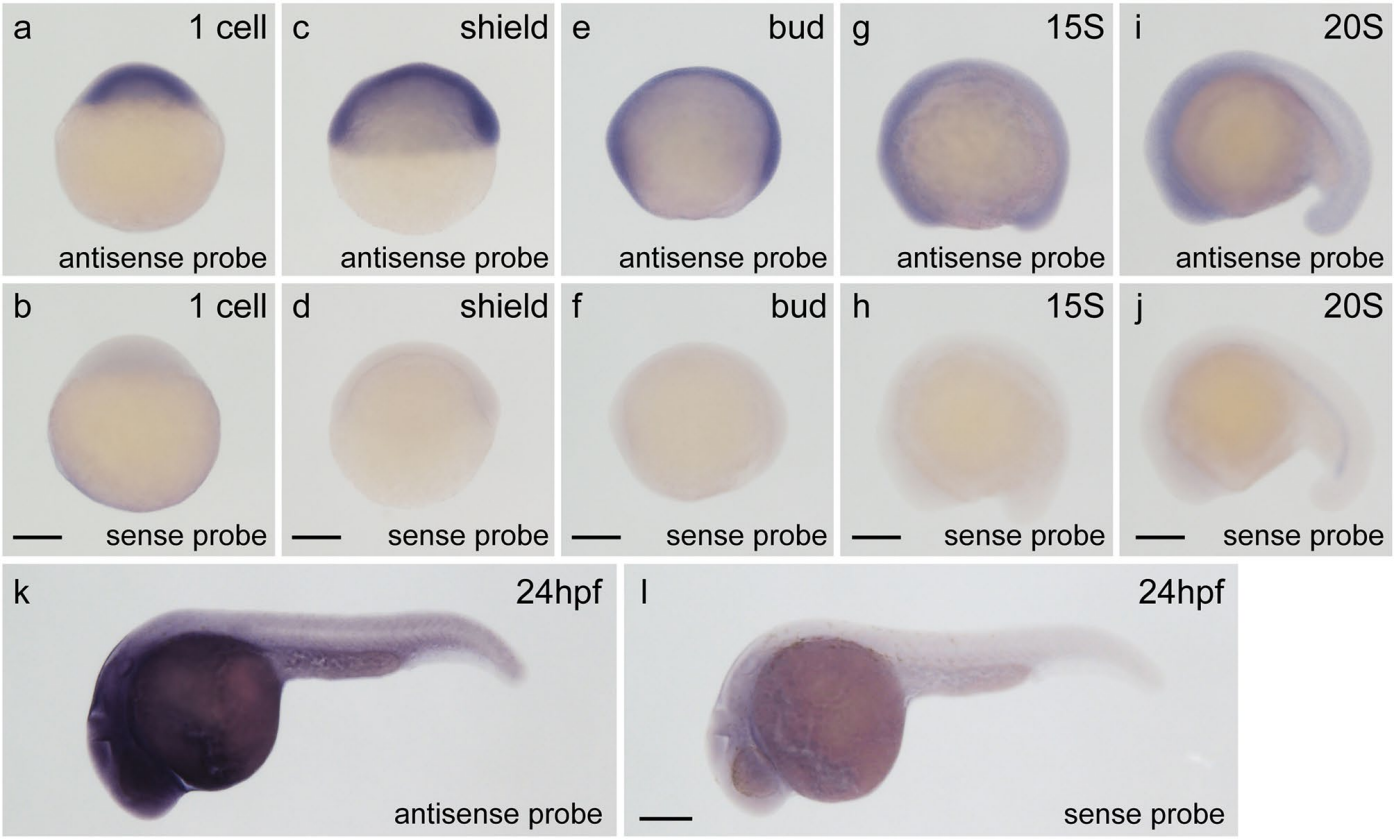
Expression of the *trrap* gene during zebrafish embryogenesis. (**a**,**b**) One-cell stage. (**c**,**d**) Shield stage. (**e**,**f**) Bud stage. (**g**,**h**) Fifteen-somite (15S) stage. (**i**,**j**) Twenty-somite (20S) stage. (**k**,**l**) Twenty-four hours post-fertilization (hpf). All pictures are lateral views. Dorsal is right (**c**). Anterior is left (**e**–**l**). The expression of the *trrap* gene was examined by whole-mount in situ hybridization (WISH) using an antisense *trrap* DIG probe (**a**,**c**,**e**,**g**,**i**,**k**) or a sense *trrap* DIG probe (**b**,**d**,**f**,**h**,**j**,**l**). Scale bar, 200 μm.

Because the developmental function of the *trrap* gene in vertebrates remains unclear, we first examined the expression pattern of the zebrafish *trrap* gene during early embryogenesis by whole-mount in situ hybridization (WISH) using an antisense *trrap* digoxigenin (DIG) RNA probe. The *trrap* mRNAs were maternally deposited at the one-cell stage and ubiquitously expressed from the shield stage to the 20-somite (20S) stage, while such a signal was not detected in the embryos incubated with the sense *trrap* DIG RNA probe (Fig. 1). The expression of *trrap* stained by the antisense *trrap* DIG RNA probe, but not the sense probe, was strongly detected in the head at 24 h post-fertilization (hpf), suggesting that the *trrap* gene is involved in craniofacial development.

Human TRRAP, which is a large protein of 3859 amino acids, possesses several functional domains, such as the FAT, PIKK-TRRAP and FATC domains^1^. The kinase domain of TRRAP (PIKK-TRRAP) lacks catalytic activity, and the functions of FAT and FATC are not fully understood. Cogné et al. reported that a cluster of human TRRAP mutations containing 13 missense variants in 24 patients was located between codons 1031 and 1159, suggesting the presence of an uncharacterized functional domain of human TRRAP. To investigate the function of the zebrafish *trrap* gene, we designed *trrap*-specific CRISPR RNAs (*trrap*-crRNA1 and *trrap*-crRNA2) targeting codon 1000 to disrupt the *trrap* gene and established *trrap*-knockout zebrafish with a 5-bp deletion that resulted in a frameshift mutation at the codon 1003 (Supplemental Fig. S1). Because the Trrap mutant protein has 1002 N-terminal amino acids and lacks most functional domains, such as the FAT, PIKK-TRRAP and FATC domains, we predict that the mutant protein is functionally disrupted.

### Morphological abnormalities in the heads of *trrap*-mutant zebrafish

Using CRISPR/Cas9 genome editing technology, Xia et al. previously established *trrap* zebrafish mutants that exhibited 7-bp deletion near the *trrap* start codon, leading to a frameshift at amino acid 7 and a premature stop codon after eight amino acids. The authors showed that the *trrap*-mutant larvae had a decreased number of deposited PLL neuromasts^6^. The predicted Trrap protein products in our *trrap* mutants had 1002 N-terminal amino acids. We did not observe these PLL neuromast defects in our *trrap* mutants at 54 hpf (Supplemental Fig. S2). In one study, most of the 13 patients with TRRAP variants in the codon 1031–1159 region (the cluster of human TRRAP mutations) had global developmental delay with craniofacial abnormalities^5^. Therefore, we examined the morphological phenotypes in the heads of the *trrap*-mutant zebrafish.

**Figure 2:**
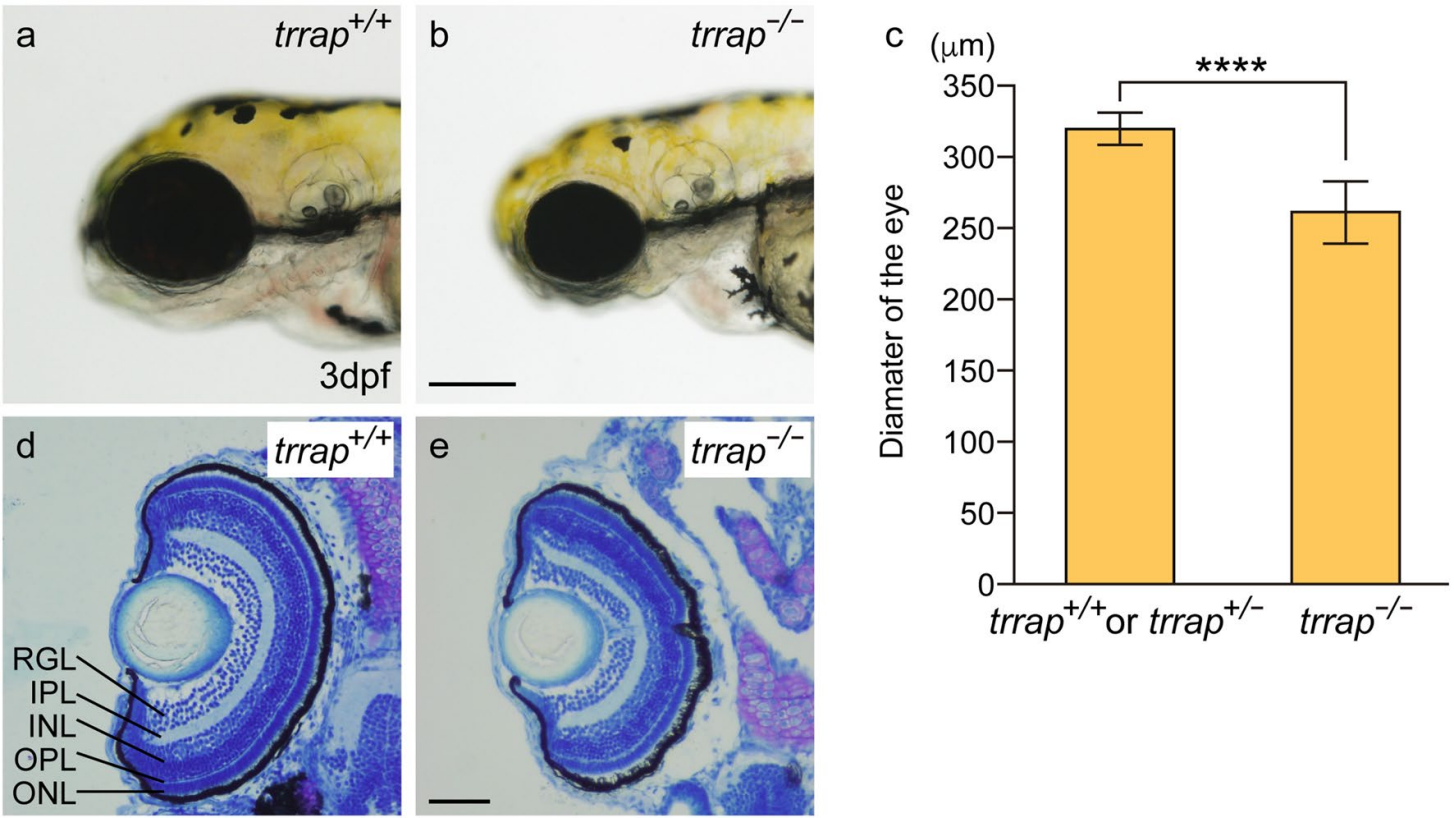
Small eyes in the *trrap*-zebrafish mutant. (**a**) Wild-type fish (*trrap*^+/+^) at 3 dpf. (**b**) *trrap* mutants (*trrap*^−/−^) at 3 dpf. Scale bar, 200 μm. (**c**) The eye diameters in larvae containing the wild-type allele (*trrap*^+/+^: n = 2; *trrap*^+/−^: n = 6) and larvae containing *trrap* mutant alleles (*trrap*^−/−^: n = 8) were measured. The error bars indicate the standard deviation. Asterisks indicate statistical significance between the wild-type and the mutant zebrafish. ****P < 0.0001. (**d**,**e**) Cross-sections of the wild-type (*trrap*^+/+^) (**d**) and the *trrap*-mutant (*trrap*^−/−^) zebrafish (**e**) at 3 dpf were stained with toluidine blue (0.1%). The eye diameter was reduced in the *trrap* mutants, whereas the laminated retinas consisting of three layers (RGL, INL and ONL) and two plexiform layers (IPL and OPL) developed normal in the wild-type and mutant zebrafish. *RGL* retinal ganglion cell layer, *IPL* inner plexiform layer, *INL* inner nuclear layer, *OPL* outer plexiform layer, *ONL* outer nuclear layer. Genomic DNA was isolated from individual caudal fins, and genotyping was performed by genomic PCR. Scale bar, 50 μm.

**Figure 3:**
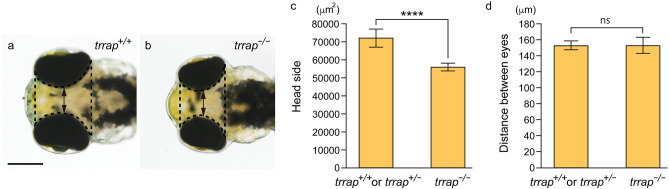
Small heads in the *trrap*-zebrafish mutant zebrafish. (**a**) Wild-type fish (*trrap*^+/+^) at 3 dpf. (**b**) *trrap* mutants (*trrap*^−/−^) at 3 dpf. Scale bar, 200 μm. (**c**) The sizes of the head excluding the eyes (dashed line) in the wild-type (*trrap*^+/+^: n = 1; *trrap*^+/−^: n = 7) and *trrap* mutant (*trrap*^−/−^: n = 8) zebrafish were measured. The error bars indicate the standard deviation. Asterisks indicate statistical significance between the wild-type and mutant zebrafish. ****P < 0.0001. (**d**) The distance between the eyes (double arrow) in the wild-type (*trrap*^+/+^: n = 1; *trrap*^+/−^: n = 7) and *trrap* mutant (*trrap*^−/−^: n = 8) zebrafish were measured. The error bars indicate the standard deviation; ns, not significant.

We observed that the diameter of the eye in the *trrap* mutants (*trrap*^−/−^: n = 8) at 3 days post-fertilization (dpf) was slightly smaller than that in the wild-type larvae containing the wild-type allele (*trrap*^+/+^: n = 2; *trrap*^+/−^: n = 6) (Fig. 2). Histological analysis using toluidine blue staining revealed that lamination of the retina to produce three nuclear layers (the retinal ganglion cell layer and the inner and outer nuclear layers) and two plexiform layers (the inner and outer plexiform layer) progressed normally in both the wild-type (*trrap*^+/+^: n = 2; *trrap*^+/−^: n = 6) and the mutant zebrafish (*trrap*^−/−^: n = 8) (Fig. 2). The area of the head was measured excluding the eyes. The size of the head was smaller in the *trrap* mutants (*trrap*^−/−^: n = 8) than in the wild-type zebrafish (*trrap*^+/+^: n = 1; *trrap*^+/−^: n = 7), while the distance between the eyes was comparable (Fig. 3).

**Figure 4:**
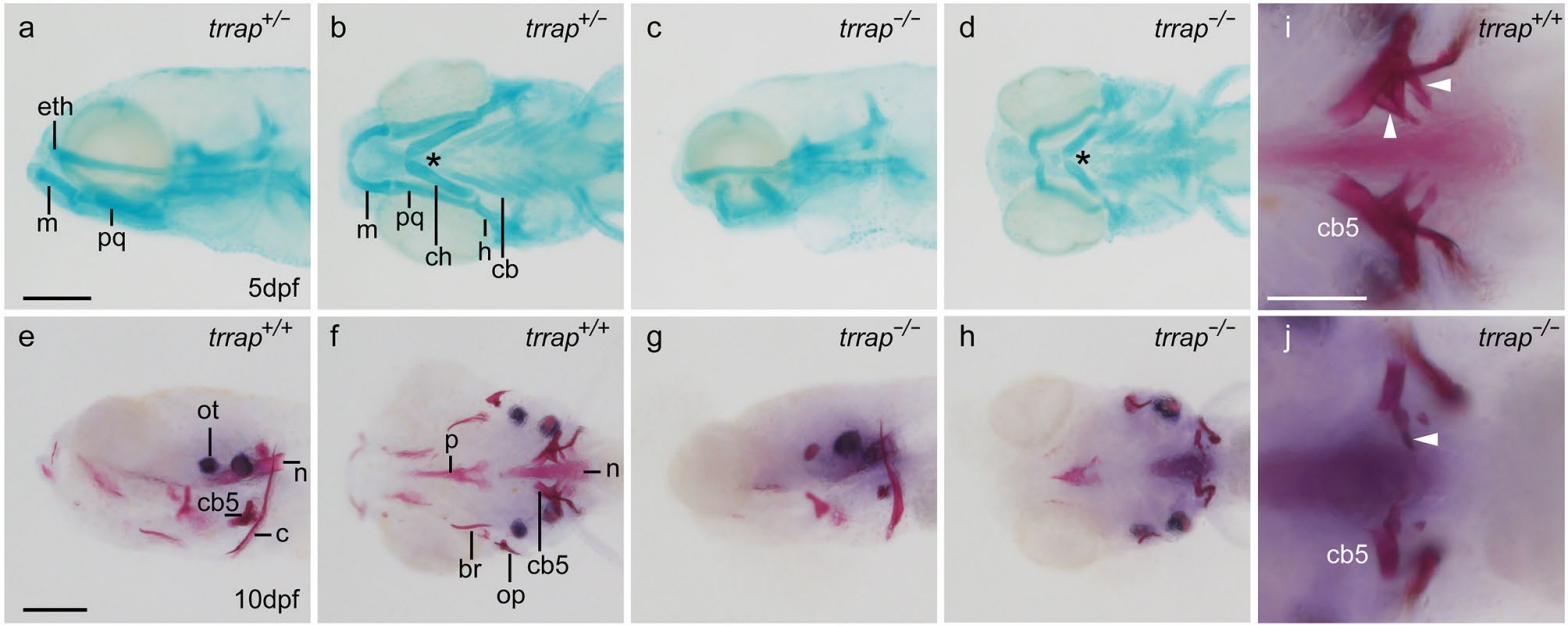
Morphological defects in the pharyngeal arches and teeth of the *trrap* mutants. (**a**–**d**) Alcian blue staining of head cartilage at 5 dpf. (**a**,**b**) Wild-type fish (*trrap*^+/−^). (**c**,**d**) *trrap* mutants (*trrap*^−/−^). (**a**,**c**) Lateral view. (**b**,**d**) Ventral view. The angle of the paired ceratohyals (indicated with an asterisk) in the *trrap* mutants was larger than that in the wild-type fish. *eth* ethmoid plate, *m* Meckel’s cartilage, *pq* palatoquadrate, *ch* ceratohyal, *h* hyosymplectic, *cb* ceratobranchials. Scale bar, 200 μm. Genomic DNA was isolated from individual fins, and genotyping of individual larvae was performed by genomic PCR. (**e**–**j**) Alizarin red staining of cranial bones at 10 dpf. (**e**,**f**,**i**) Wild-type fish (*trrap*^+/+^). (**g**,**h**,**j**) *trrap* mutant (*trrap*^−/−^). (**e**,**g**) Lateral view. (**f**,**h**–**j**) Ventral view. The white arrowheads indicate mineralized teeth (**i**), whereas tooth mineralization was diminished in the *trrap* mutants (**j**). *ot* otolith, *n* notochord, *cb5* ceratobranchial 5, *c* cleithrum, *p* parasphenoid, *br* branchiostegal rays, *op* opercle. Scale bar, 200 μm (**e**–**h**). Scale bar, 100 μm (**i**,**j**). Genomic DNA was isolated from individual fins, and genotyping of individual larvae was performed by genomic PCR.

Next, we examined the morphologies of the pharyngeal arches using Alcian blue to stain sulfated and carboxylated acid mucopolysaccharides^9^. We found that the length of ceratohyal cartilage was reduced in the mutants at 5 dpf (Fig. 4 and Table 1). The angle of the paired ceratohyals in the mutants was larger than that in the wild-type fish, whereas the morphologies of the ethmoid plate and ceratobranchials appeared to be normal in the mutants (Fig. 4 and Table 1).

**Table 1:** Ceratohyal length and angle in wild-type and *trrap*-mutant zebrafish.

	Wild-type	*trrap* mutant
Ceratohyal length (μm)	223.75 ± 15.59	150 ± 11.55
Ceratohyal angle (°)	60.62 ± 7.3	95.63 ± 16.78

The ceratohyal length and angle of in wild-type (n = 8) and *trrap*-mutant (n = 8) zebrafish were measured, and the numerical values represent the means ± SDs (standard deviations).

Furthermore, we examined bone formation in the head using Alizarin red to stain calcium deposits in tissues^9^. We found that ossification hypoplasia was present in the head but not in cleithrum in the *trrap* mutants (*trrap*^−/−^: n = 14) compared to the wild-type fish (*trrap*^+/+^: n = 5; *trrap*^+/−^: n = 11) (Fig. 4). Notably, wild-type fish possessed mineralized teeth on ceratobranchial 5, whereas the *trrap* mutants exhibited impairment of tooth mineralization. We observed similar defects in eyes, head, ventral pharyngeal arches and tooth development (Supplemental Fig. S3), when other *trrap* crRNAs (*trrap*-crRNA3 and *trrap*-crRNA4) were injected with tracrRNA and Cas9 into zebrafish embryos in the one-cell stage. These results suggest that the *trrap* gene is required for appropriate craniofacial development, including the development of the eyes, head, ventral pharyngeal arches and teeth.

### Expression of pharyngeal arch and tooth marker genes in the *trrap* mutant

**Figure 5:**
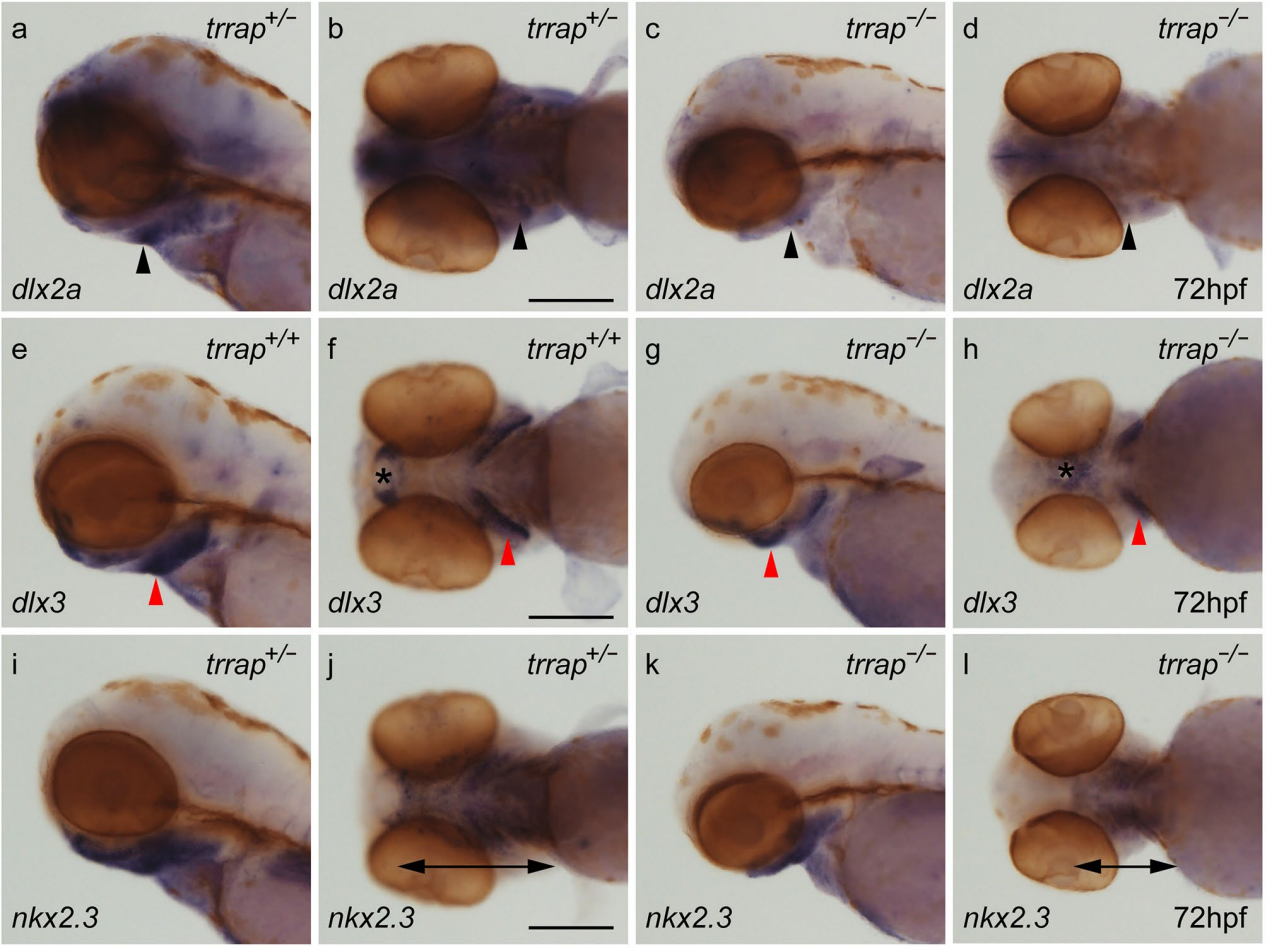
Differential expression of pharyngeal arch genes in the *trrap* mutants. Whole-mount in situ hybridization (WISH) analysis for *dlx2a* (**a**–**d**), *dlx3* (**e**–**h**), and *nkx2.3* (**i**–**l**) at 72 hpf. (**a**,**c**,**e**,**g**,**i**,**k**) Lateral views. (**b**,**d**,**f**,**h**,**j**,**l**) Ventral views. All pictures show the anterior aspect to the left. The expression of *dlx2a* (black arrowheads), *dlx3* (red arrowheads) and *nkx2.3* (double arrows) in the ventral pharyngeal arches was narrowed and restricted in the *trrap* mutants. The asterisks indicate the position of the mouth. After images were taken, genomic DNA was isolated from individual larvae, and genotyping was performed by genomic PCR. Scale bar, 200 μm.

Because the *trrap* mutants exhibited craniofacial abnormalities, including pharyngeal arch and tooth defects, we investigated the expression of pharyngeal arch (*dlx2a*, *dlx3* and *nkx2.3*) and tooth (*dlx2b* and *pitx2*) marker genes by WISH. The *dlx2a* is expressed in the pharyngeal arch ectomesenchyme^10^ and the *dlx3* is expressed in the ventral mesenchymal cells^11^, while the *nkx2.3* is expressed in the lateral pharyngeal endoderm^12^. The expression patterns of pharyngeal arch genes in the *trrap* mutants were similar to those of wild-type zebrafish at 24 hpf (Supplemental Fig. S4). The expression of *dlx2a*, *dlx3* and *nkx2.3* at 72 hpf was narrowly restricted in the pharyngeal arches of the mutants compared to those of the wild-type zebrafish (Fig. 4). The *dlx2b* and *pitx2* genes at 72 hpf were weakly expressed in the developing teeth of the mutants (Fig. 5). The expression levels of neural genes, *elavl3/huC* (neuron), *gfap* (radial glial and neural progenitors), *olig2* (primary motor neuron and oligodendrocyte) and *gli2a* (central nervous system and pharyngeal arch), in the central nervous system (CNS) at 54 hpf were comparable between the wild-type and the *trrap*-mutant zebrafish (Supplemental Fig. S5). These results suggest that the expression of pharyngeal arch and tooth marker genes was restricted and diminished in the *trrap* mutant, respectively.

## Discussion

Recent accumulating evidence demonstrates that human patients with genetic mutations in the *TRRAP* gene have various symptoms, including facial dysmorphisms, microcephaly, global developmental delay and intellectual disability^5^. Because the *trrap*-mutant zebrafish exhibited tooth hypoplasia, small eyes and head, and short ventral pharyngeal arches (Figs. 2, 3, 4), we proposed that the functional impairment of the human *TRRAP* and zebrafish *trrap* genes causes craniofacial abnormalities, including microcephaly.

Recently, Xia W. et al. reported that the p.Arg171Cys variant near the T-terminus of the human TRRAP cosegregated with hearing loss^6^. They established *trrap*-knockout zebrafish that contained 7-bp deletion near the start codon, leading to a frameshift at amino acid 7 and a premature stop codon after eight amino acids. Their *trrap*-mutant zebrafish exhibited decreased numbers of posterior lateral line (PLL) neuromasts and an impaired acoustic startle response (ASR), however, they did not mention other morphological abnormalities in the mutants. Cogné et al. independently reported that most patients with missense *TRRAP* variants exhibited primarily facial dysmorphisms, global developmental delay and intellectual disability^5^. Hearing impairment (3/24 individuals) and visual impairment (4/24 individuals) were observed in a small proportion of the patients. They also found that the cluster of human TRRAP mutations containing 13 variants carried by 24 patients was located between codons 1031 and 1159. In this study, we established a zebrafish *trrap* mutant that presumably generated the 1002 N-terminal amino acids. We found that our *trrap*-mutant zebrafish exhibited several defects in craniofacial development, but the mutants had normal numbers of PLL neuromasts. It is not clear why the two different *trrap* mutant alleles show different phenotypes in zebrafish. Because their *trrap* mutant zebrafish has a premature stop codon near the start codon^6^, there is a possibility of the presence of *trrap* transcripts from the second methionine codon (18–3841 amino acids). In the case of *tif1γ*/*moonshine* (*mon*) mutant zebrafish, the hypomorphic phenotype in the *mon*^*m262*^ allele is interpreted by the presence of N-terminal truncated Tif1γ protein from another methionine downstream of a premature stop codon^13^. The patients with different missense mutations in human *TRRAP* gene exhibit various symptoms, including facial dysmorphisms and microcephaly, therefore, further analysis is required to clarify what kind molecules associate with the uncharacterized domain of TRRAP (1031–1159 amino acids).

The expression of the zebrafish *trrap* gene was detected in the developing head, including in the eyes and pharyngeal arches, during early development (Fig. 1). We found that the eye diameter was slightly reduced in the *trrap* mutants at 3 dpf, whereas lamination of the retina to produce three nuclear layers and two plexiform layers occurred in the mutants (Fig. 2). The *trrap* mutants exhibited small heads compared to the wild-type fish, while the distance between the eyes was comparable (Fig. 3). The angle of the paired ceratohyal cartilage was larger than that of the wild-type ceratohyal cartilage (Fig. 4). We observed that the mineralization of teeth on ceratobranchial 5 was inhibited in the *trrap* mutants, while the wild-type fish had normally mineralized teeth. We confirmed that *trrap*-crispants injected with *trrap*-crRNA3, *trrap*-crRNA4, tracrRNA and Cas9 developed tooth hypoplasia, small eyes and head, and short ventral pharyngeal arches. Thus, these morphological abnormalities in the head may be associated with the craniofacial malformations of patients with *TRRAP* mutations^5^.

**Figure 6:**
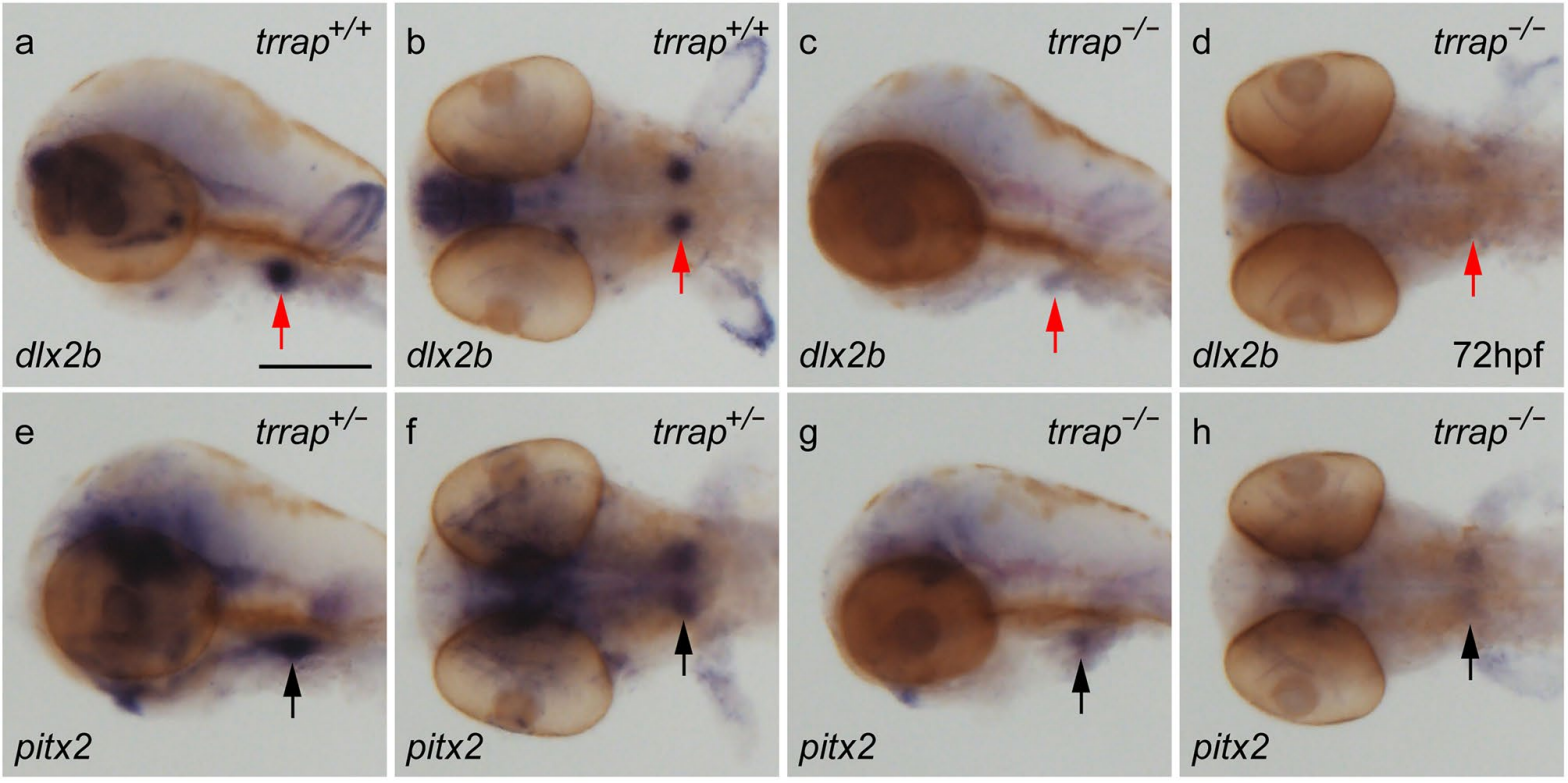
Differential expression of tooth marker genes in the *trrap* mutants. WISH analysis for *dlx2b* (**a**–**d**) and *pitx2* (**e**–**h**) at 72 hpf. (**a**,**c**,**e**,**g**) Lateral views. (**b**,**d**,**f**,**h**) Ventral views. All pictures show the anterior aspect to the left. The expression of *dlx2b* (red arrow) and *pitx2* (black arrow) in the developing teeth was weak in the mutants. After images were taken, genomic DNA was isolated from individual larvae, and genotyping was performed by genomic PCR. Scale bar, 200 μm.

The contribution of Trrap to craniofacial development may be dependent on the activity of the HAT complex. Because the inhibition of histone deacetylation is involved in neural differentiation^2^, the ability to make the chromatin structure acceptable for transcriptional activation is an important aspect of targeted gene activation. One possible explanation is that the craniofacial defects in the *trrap* mutants were due to insufficient maintenance of pharyngeal arch gene expression and insufficient activation of tooth genes. We found that the expression patterns of pharyngeal arch markers (*dlx2a*, *dlx3* and *nkx2.3*) at 24 hpf were similar to those in the wild-type, but these expression patterns were narrowly restricted at 72 hpf (Fig. 5, Supplemental Fig. S4). The expression of tooth markers (*dlx2b* and *pitx2*) was diminished in the *trrap* mutants (Fig. 6). On the other hand, the expression patterns of neural genes, such as *elavl3/huC*, *gfap*, *olig2* and *gli2a*, were comparable in the CNS in the wild-type and mutant zebrafish. Possible targeted genes regulated by Trrap may be required for appropriate craniofacial development of structures including the head, eye, pharyngeal arches and teeth. We cannot exclude the possibility that the transcriptional difference might have been caused by dysfunction other than impaired HAT activity. Further analysis will be required to clarify how the Trrap protein contributes to craniofacial development in vertebrates. Because *trrap*-mutant zebrafish partly recapitulate craniofacial malformations, including microcephaly, observed in patients with genetic *TRRAP* mutations, we propose that *trrap*-mutant zebrafish are a useful disease model for human *TRRAP* gene mutations.

## Methods

### Zebrafish maintenance and ethics statement

One-year-old adult heterozygous *trrap*-mutant zebrafish were maintained in a controlled aquatic facility with purified water by a reverse osmosis system with the following conditions: 14/10 h light/dark photoperiod, 28.5 °C (± 1 °C), pH 7.0 (± 1) and conductivity 450 mS/cm. Zebrafish embryos were obtained from mating of adult heterozygous *trrap*-mutant zebrafish. The collected embryos were washed with E3 water, and fertilized embryos were selected with an optical microscope (Olympus SZ61). All animal experiments were performed in accordance with institutional and national guidelines and regulations. The study was carried out in compliance with the ARRIVE guidelines^14^. The study was approved by the Institutional Animal Care and Use Committee of the University of Yamanashi (Approval Identification Number: A30-25).

### Genome editing for the *trrap* locus

We used a ready-to-use CRISPR/Cas9 system with CRISPR RNA (crRNA), *trans-activating* crRNA (tracrRNA) and recombinant Cas9 protein to disrupt the zebrafish *trrap* gene^15^. Synthetic crRNAs and tracrRNA (Supplementary Table S1) and recombinant Cas9 protein were obtained from Integrated DNA Technologies, Inc. (IDT). Synthetic *trrap*-crRNA1 (25 pg), *trrap*-crRNA2 (25 pg) and tracrRNA (100 pg) were injected with recombinant Cas9 protein (1 ng) into one-cell-stage zebrafish embryos. To confirm the phenotypes of the *trrap* mutants, synthetic *trrap*-crRNA3 (25 pg), *trrap*-crRNA4 (25 pg) and tracrRNA (100 pg) were injected with recombinant Cas9 protein (1 ng) into one-cell-stage zebrafish embryos.

### Genotyping of the zebrafish *trrap* mutants

Zebrafish embryos and larvae at the indicated stages were incubated in 108 μl of 50 mM NaOH at 98 °C for 10 min to isolate the genomic DNA. Subsequently, 12 μl of 1 M Tris–HCl (pH 8.0) was added to the solution^16^. The targeted genomic fragments were amplified by PCR with PrimeTaq (Primetech) using the locus-specific primers listed in Supplementary Table S2. The PCR conditions were as follows: 40 cycles of 98 °C for 10 s, 55 °C for 30 s and 72 °C for 30 s. For the heteroduplex mobility assay (HMA), the resultant PCR amplicons were electrophoresed on 12.5% polyacrylamide gels^15^.

### Alcian blue staining and Alizarin red staining

Fixed larvae at 5 dpf were incubated overnight with 4% paraformaldehyde. After three washes with phosphate-buffered saline (PBS) containing 0.1% Tween 20 (PBS-T), the larvae were dehydrated with ethanol and incubated overnight with Alcian blue (0.02%) in 30% acetic acid and 70% ethanol^17^. The larvae were rehydrated with ethanol and incubated with 2% KOH and subsequently incubated with bleaching buffer (1% H_2_O_2_ and 1% KOH) for 1 h. The larvae were washed three times with 2% KOH.

Alizarin red staining was performed as described previously^17^. Larvae at 10 dpf were incubated with 4% paraformaldehyde in PBS and washed twice with PBS-T. The larvae were incubated with bleaching buffer for 30 min and washed with PBS-T twice. After 1 h of incubation with 1 mg/ml Alizarin red in 0.5% KOH at room temperature, the larvae were washed with 0.5% KOH.

### Histological analysis

Fixed larvae were incubated in ethanol and embedded using a Technovit 8100 kit (Kulzer). The embedded larvae were sectioned at 5 μm on a Leica RM2125 microtome and mounted on slides. The larvae were stained with toluidine blue (0.1%) after sectioning^18^.

### Whole-mount in situ hybridization (WISH)

The expression of *trrap*, *dlx2a*^19^, *dlx2b*, *dlx3*^11^, *nkx2.3*^12^, *pitx2*, *elavl3*^20^, *gfap*, *olig2*^21^ and *gli2a* was examined by WISH as previously described^22^. Zebrafish embryos and larvae hybridized with the digoxygenin (DIG)-labeled antisense RNA probe were incubated with an alkaline phosphatase-conjugated anti-DIG antibody. The embryos and larvae were incubated with BM Purple (Roche) as the substrate to visualize the RNA probe recognized by the anti-DIG antibody. After three washes with PBST, the embryos and larvae were incubated with 4% paraformaldehyde.

### Lateral line neuromast labeling

Larvae at 54 hpf were fixed in 4% paraformaldehyde for 3 h at room temperature and washed with PBS-T three times. The larvae were incubated in alkaline phosphatase buffer containing NBT and BCIP (Nacalai Tesque) for 30 min.

## Supplementary Information

Supplementary Information.
