# Supplementary material for: Involvement of the zebrafish trrap gene in craniofacial development
Source: Sci Rep. 2021 Dec 21;11:24166. doi: 10.1038/s41598-021-03123-z (PMC8692476; doi:10.1038/s41598-021-03123-z)
Supplement: Supplementary file 1 — Supplementary Information. [file 41598_2021_3123_MOESM1_ESM.pdf]

Involvement of the zebrafish *trrap* gene in craniofacial development

Taichi Suzuki[1], Yo Hirai[1], Tomoko Uehara[2], Rie Ohga[1], Kenjiro Kosaki[2] & Atsuo Kawahara[1*]

[1]Laboratory for Developmental Biology, Graduate School of Medical Science, University of Yamanashi, 1110 Shimokato, Chuo, Yamanashi, 409-3898, Japan

[2]Center for Medical Genetics, Keio University School of Medicine, 35 Shinanomachi, Shinjuku-ku, Tokyo, 160-8582, Japan

*Correspondence should be addressed to Atsuo Kawahara (akawahara@yamanashi.ac.jp).

Supplemental Figures

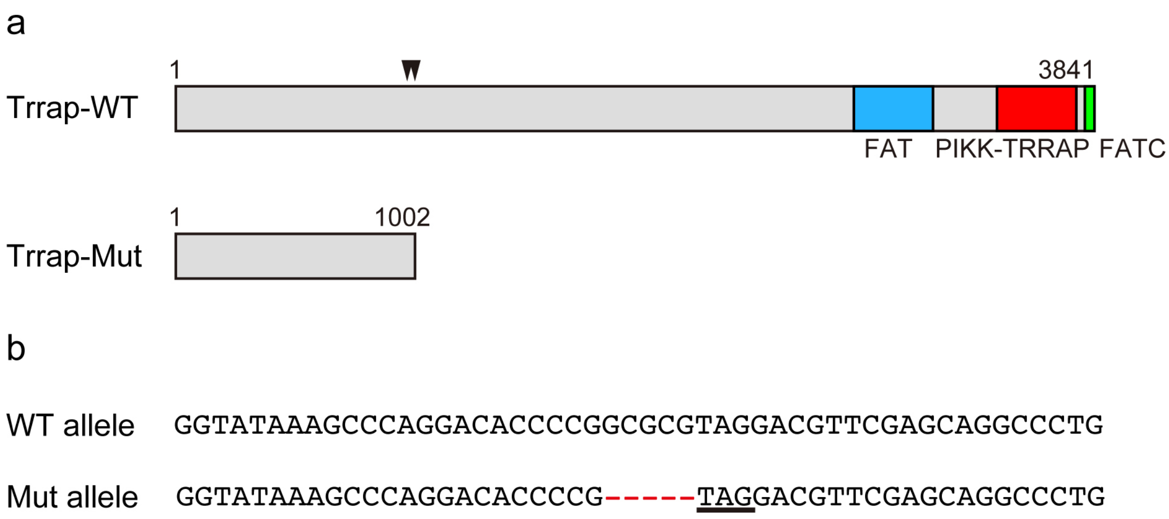

Figure S1. Predicted molecular structures of the wild-type and mutant Trrap proteins. (a) Mutations in the *trrap* gene cause a premature stop codon (as indicated by an underline in Figure S1b) at the codon 1,003. The FAT (FRAP, ATM, TRRAP) domain is indicated by the blue rectangle. The PIKK-TRRAP (the pseudokinase domain of TRRAP) is indicated by the red rectangle. The FATC (FRAP, ATM, TRRAP C-terminal) domain is indicated by the green rectangle. (b) CRISPR/Cas9-induced mutation in the *trrap* gene is shown in the annotated mutant sequence. The red dashes (–) are deleted nucleotides (5-bp deletion).

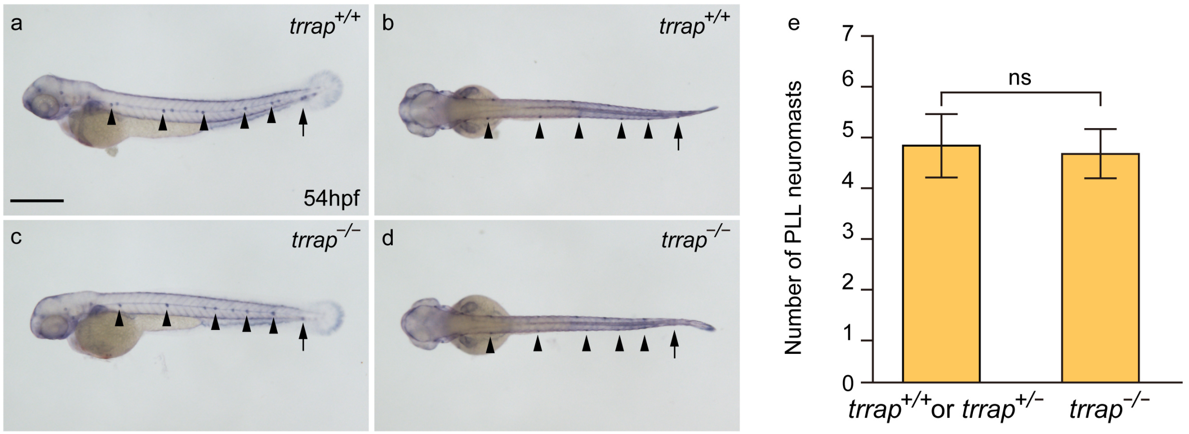

Figure S2. Posterior lateral line (PLL) neuromast deposition at 54 hpf. (a, b) Wild-type fish (*trrap*$^{+/+}$) at 54 hpf. (c, d) *trrap* mutant (*trrap*$^{-/-}$) at 54 hpf. (a, c) Lateral views. (b, d) Ventral views. (a-d) Alkaline phosphatase accumulation in lateral line neuromasts. The arrowheads indicate the positions of PLL neuromasts on the left side. The arrows indicate the positions of terminal neuromasts. (e) The number of PLL neuromasts were quantified in the wild-type (*trrap*$^{+/+}$: n=4; *trrap*$^{+/-}$: n=4) and the mutant fish (*trrap*$^{-/-}$: n=7). The error bars indicate the standard deviation; ns, not significant. After images were taken, genomic DNA was isolated from individual larvae, and genotyping was performed by genomic PCR. Scale bar, 0.5 mm.

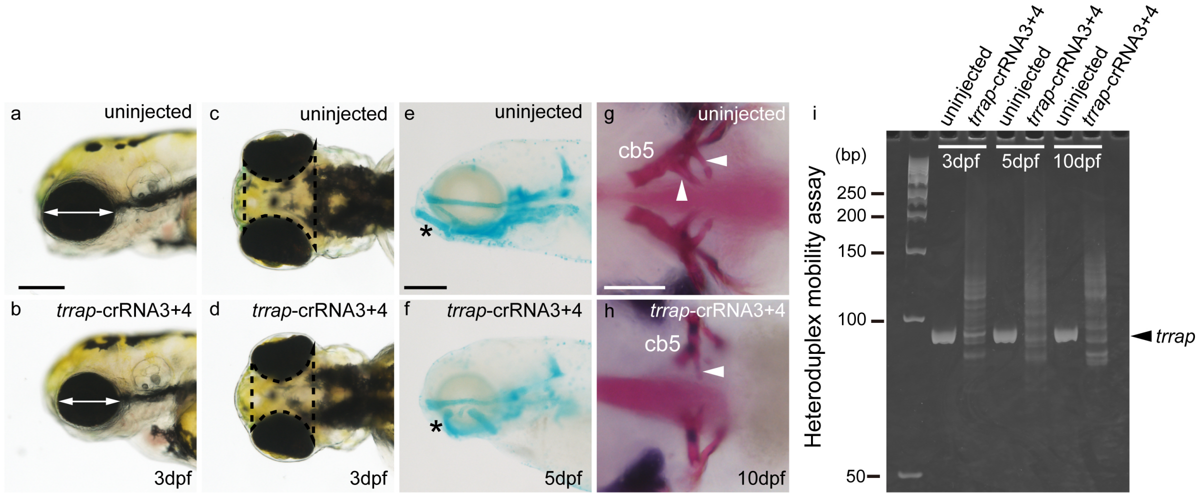

Figure S3 Morphological abnormalities of the *trrap*-crispant larvae. (a, b) The eyes of wild-type (a) and *trrap*-crispant larvae (b) at 3 dpf (lateral view). (c, d) The heads of wild-type (c) and *trrap*-crispant larvae (d) at 3 dpf (dorsal view). (e, f) Alcian blue staining of pharyngeal arches of wild-type (e) and *trrap*-crispant larvae (f) at 5 dpf (lateral view). (g, h) Alizarin red staining of cranial bones of wild-type (g) and the *trrap*-crispant larvae (h) at 10 dpf (ventral view). Scale bar, 200 μm (a-f). Scale bar, 100 μm (g, h). Synthetic *trrap*-crRNA3 (25 pg), *trrap*-crRNA4 (25 pg) and tracrRNA (100 pg) were injected with recombinant Cas9 protein (1 ng) into one-cell-stage zebrafish embryos. The head (dashed line), eyes (double arrow) and Meckel's cartilage (asterisk) in *trrap*-crispant larvae were smaller than those in wild-type (a-f). The mineralization of teeth (arrowheads) was impaired in the *trrap*-crispant (g, h). (i) Genomic mutations in the targeted *trrap* locus. The targeted-*trrap* fragments (arrowhead: 92 bp) were amplified by PCR and electrophoresed on 12.5% polyacrylamide gel. Various indel mutations were detected in the targeted *trrap* locus. After images were taken, genomic DNA was isolated from individual larvae, and indel mutations in the targeted *trrap* locus were determined by heteroduplex mobility assay (HMA).

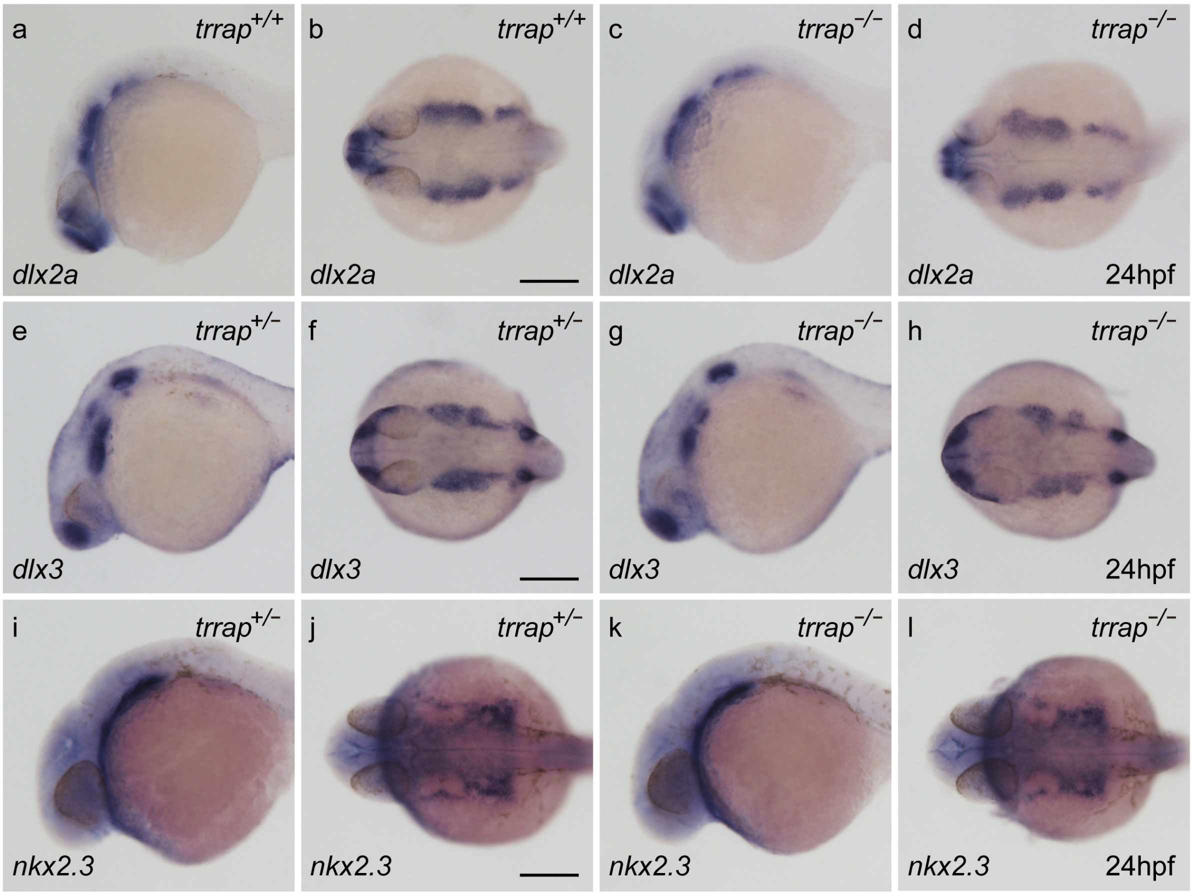

Figure S4 Expression of pharyngeal arch genes in the *trrap* mutants. (a-l) WISH analysis for *dlx2a* (a-d), *dlx3* (e-h) and *nkx2.3* (i-l) at 24 hpf. (a, c, e, g, i, k) Lateral views. (b, d, f, h, j, l) Ventral views. All pictures show the anterior aspect to the left. The expression of *dlx2a*, *dlx3* and *nkx2.3* in the developing pharyngeal arches was comparable between the wild-type and the *trrap* mutant zebrafish. After images were taken, genomic DNA was isolated from individual eambryos, and genotyping was performed by genomic PCR. Scale bar, 200 μm.

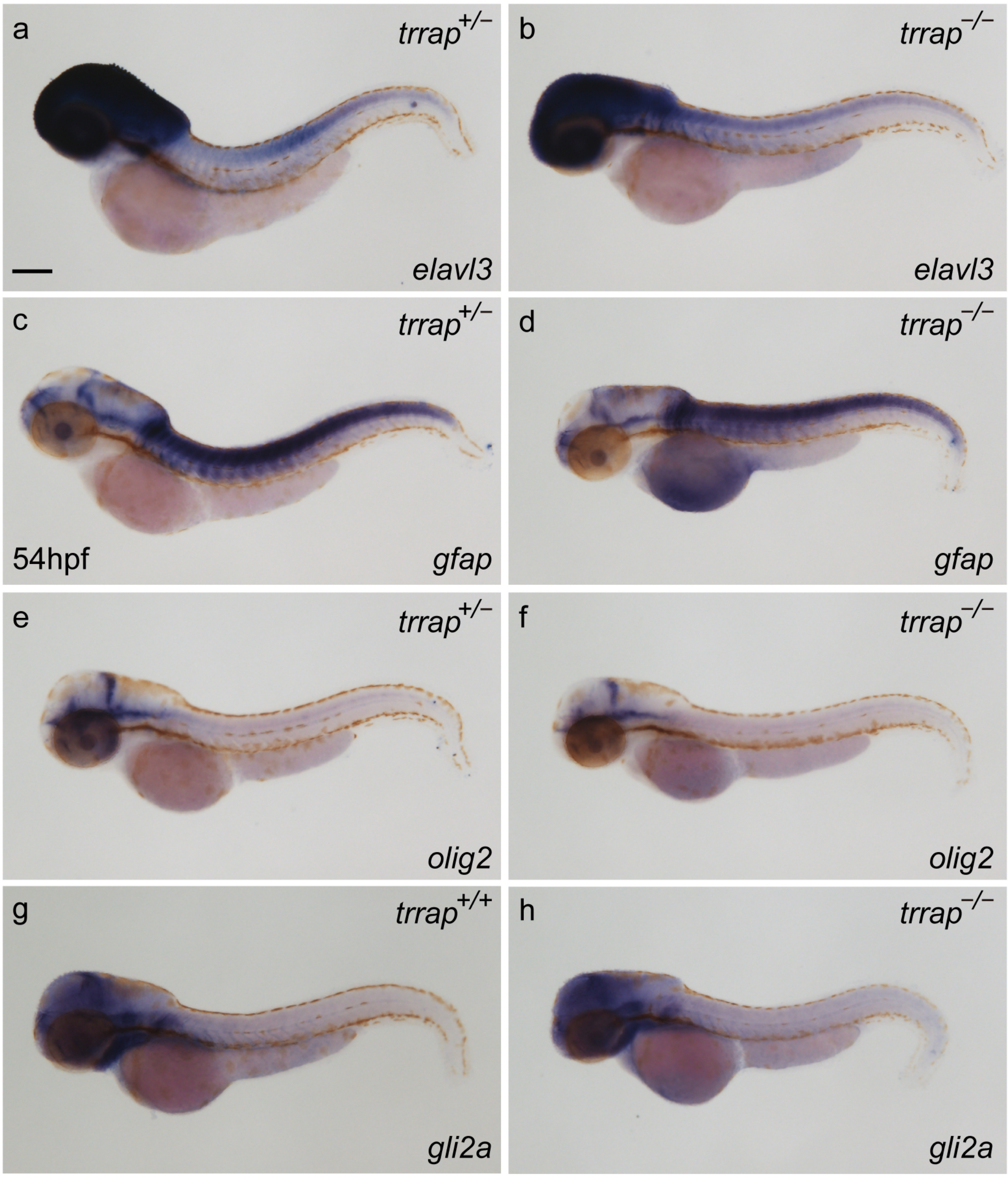

Figure S5 Expression of neural genes in the *trrap* mutants. (a-h) WISH analysis for *elavl3* (a, b), *gfap* (c, d), *olig2* (e, f) and *gli2a* (g, h) at 54 hpf. (a-h) All pictures show the anterior aspect to the left (lateral views). The expression levels of *elavl3*, *gfap*, *oligo* and *gli2a* in the CNS were comparable between the wild-type and the *trrap* mutant zebrafish. After images were taken, genomic DNA was isolated from individual larvae, and genotyping was performed by genomic PCR. Scale bar, 200 μm.

Supplemental Table

Table S1 Targeted genomic sequences.

Targeted genomic sequences for CRISPR/Cas9

Target	Sequence (5' to 3')
trrap-1	CTCGAACGTCCTACGCGCCGGGG
trrap-2	AGACATGAACGCTCCTGTCAGGG
trrap-3	CCGCCATTACACCATGGTGGCGG
trrap-4	CGGATCAAGCTGGCTACAAACGG

PAM sequences are underlined.

Table S2 PCR primers used in this study.

Primer name	Sequence (5' to 3')
trrap-HMA-F1	ATCTCCCACCGGTATAAAGC
trrap-HMA-R1	GGCCTGAGATCCTTGATGAC
trrap-HMA-F2	CAAGGATCTCAGGCCCAGCG
trrap-HMA-R2	ACTACTCACCACACTGTTGAG
